# Use of healthcare services and prescription medication prior to sarcoma diagnosis in children, adolescents, and young adults in 1997–2020: a population-based cohort study

**DOI:** 10.1007/s10552-025-02077-1

**Published:** 2025-10-09

**Authors:** Daniel Thor Halberg Dybdal, Ólafur Birgir Davidsson, Signe Holst Søegaard, Michael Mørk Petersen, Ninna Aggerholm-Pedersen, Henrik Hjalgrim, Klaus Rostgaard, Lisa Lyngsie Hjalgrim

**Affiliations:** 1 Danish Cancer Institute, Danish Cancer Society, Copenhagen, Denmark; 2 Department of Paediatric and Adolescent Medicine, Copenhagen University Hospital Rigshospitalet, Copenhagen, Denmark; 3 Department of Congenital Disorders, Statens Serum Institut, Copenhagen, Denmark; 4 Department of Clinical Medicine, University of Copenhagen, Copenhagen, Denmark; 5 Department of Orthopaedic Surgery, Copenhagen University Hospital Rigshospitalet, Copenhagen, Denmark; 6 Department of Oncology, Aarhus University Hospital, Aarhus, Denmark; 7 Department of Hematology, Copenhagen University Hospital Rigshospitalet, Copenhagen, Denmark

**Keywords:** Sarcoma, Diagnostic interval, Children, Adolescents, Young adults

## Abstract

**Purpose:**

Sarcomas are among the leading causes of cancer death in children, adolescents, and young adults and survival has not been substantially improved for decades. Reducing the diagnostic interval could contribute meaningfully to increased survival at a low- cost. This study provided much-needed knowledge of the prediagnostic patient trajectories to direct future clinical efforts.

**Methods:**

This population-based study examined the use of healthcare services in the two years preceding diagnosis in 1524 children, adolescents, and young adults with sarcoma compared to the background population, based on Danish national register data from 1997 to 2020.

**Results:**

Sarcoma patients were more likely than the background population to have contacts in the primary healthcare sector, hospital outpatient clinics, and emergency rooms, and to fill prescriptions for pain- and antimicrobial medication for up to 24 consecutive months before diagnosis.

Patients with metastatic sarcoma at the time of diagnosis were more likely than non-metastatic patients to use healthcare services in the last 2–4 months prior to diagnosis.

**Conclusion:**

Reducing the diagnostic interval in early-life sarcomas should be explored as a low-cost complement to new, advanced treatments to improve survival. Efforts should focus on improving the selection of CAYA with musculoskeletal symptoms for early referral and a better understanding of the diagnostic pathway within the secondary healthcare system.

**Supplementary Information:**

The online version contains supplementary material available at 10.1007/s10552-025-02077-1.

## Background

Cancer remains the leading disease-related cause of death in children, adolescents, and young adults (CAYA) living in affluent settings. Sarcomas of bone- and soft-tissue are, respectively, the third and fourth leading cause of cancer death in CAYA, accounting for 21% of cancer deaths in these age groups [1]. Compared to other early-life cancers, survival rates from sarcomas are generally poor and have not seen significant improvements in several decades [2–6].

Among the strongest predictors of survival from sarcoma are metastatic stage and tumor size at the time of diagnosis – both markers of biologically advanced disease [3, 4, 6–8]. While the relation of these characteristics to the length of the diagnostic interval (the interval from first clinical contact to diagnosis) is not clearly understood, it is a reasonable assumption that reducing the diagnostic interval would contribute meaningfully to increased survival [9]. However, early referral/diagnosis of sarcomas is challenging due to their rarity, and their often insidious and non-specific onset symptoms. In other early-life cancers, a reduction in the diagnostic interval was attainable with low-cost measures, independent of the need for new diagnostic modalities. For example, studies of prediagnostic trajectories of brain cancers in children and adolescents in the United Kingdom led to a national campaign, *HeadSmart*, which halved the median total diagnostic interval and the time from first medical contact to relevant imaging [10].

Applying a similar approach in early-life sarcomas is desirable. However, comprehensive knowledge of the prediagnostic trajectories of CAYA sarcoma patients is lacking. This is primarily due to the rarity of these cancers and the lack of consistent, population-wide data on relevant healthcare services in most countries. Among the few studies specifically investigating use of healthcare services prior to a sarcoma diagnosis, most were limited to selected adult populations, and none included pre-adolescent children [11–13]. Conversely, studies of prediagnostic health behaviors in children and adolescents with cancer have rarely reported separate results for sarcoma patients [14, 15]. A population-based study of cancer patients aged 15–39 years found that the incidence rate ratio for face-to-face contacts with a general practitioner (GP) was increased starting five months before diagnosis in bone sarcoma patients and 12 months before diagnosis in soft-tissue sarcoma patients, compared with matched controls [16].

Most previous studies were limited in scope, either in terms of healthcare events included or the length of the prediagnostic period covered. Some studies described temporal patterns in healthcare use among sarcoma patients but lacked comparisons to background populations [12], while others focused on describing the prevalence and duration of symptoms, but did not relate these to healthcare use [13, 14].

While national referral guidelines and fast-track cancer patient pathways (CPPs) have improved patient trajectories for various cancers in multiple countries, guidelines for early-life sarcomas remain limited. For example, the UK National Institute for Health Care Excellence guidelines on the recognition and referral of suspected cancer include only one pre-imaging recommendation each for bone- and soft-tissue sarcoma in children and young people [17]: An X-ray within 48 h for unexplained bone swelling or pain, and an ultrasound within 48 h for an unexplained soft-tissue mass increasing in size. The British Sarcoma Group guidelines also include nighttime pain as a red flag symptom for bone tumors [18]. The Danish Health Authority’s CPPs for pediatric cancers and sarcomas (the latter is not age-specific) both recommend considering bone cancer in patients with a palpable bony mass or persistent, unexplained bone or joint pain [19, 20]. Only the pediatric CPP specifies *persistent* (as lasting more than 1–2 weeks). Neither CPP mentions nighttime pain, nor do they specify the urgency/timeline of referral for first imaging.

Overall, there is a lack of comprehensive, population-based evidence to inform prediagnostic strategies for suspected sarcoma in CAYA. Reducing the diagnostic interval in these patients requires a better understanding of their health-seeking behaviors from symptom onset to diagnosis. To address this knowledge gap, we used Danish population-wide registers to answer two key questions:

i) Are CAYA with sarcoma more likely than matched peers without sarcoma to use selected healthcare services in the 24 months preceding diagnosis? If so, for how many consecutive months prior to diagnosis is there a difference?

ii) Do CAYA sarcoma patients with (M1-stage) and without (M0-stage) metastatic disease at diagnosis have different patterns of healthcare services use prior to diagnosis, when compared to matched peers without sarcoma?

## Methods

### Study population

Using the Danish Cancer Register, we identified all individuals in Denmark with an incident diagnosis of sarcoma before 40 years of age from 1997 to 2020. Sarcoma diagnoses were identified by morphology codes according to the International Classification of Diseases for Oncology, 3rd Edition (ICD-O-3 [21]. While there is no universal definition of young adulthood, there is precedence in cancer research and in clinical guidelines for an inclusive definition with an upper age-limit of 39 years, as used by the European Society for Medical Oncology (ESMO) and the US National Cancer Institute (NCI) [22, 23].

For each sarcoma proband, we randomly sampled ten comparators among eligible persons in the background population. Eligibility was defined as not being diagnosed with a sarcoma, matching on sex, month of birth and municipality of residence, and being alive and living in Denmark at the proband’s date of diagnosis. Each comparator was assigned a surrogate diagnosis date identical to the diagnosis date of their matched proband.

Assuming that age affects an individual’s use of healthcare services, we divided the study population into three age groups, based on the World Health Organization (WHO) definition of adolescence: Pre-adolescent children (aged 0–9 years), adolescents (aged 10–19 years), and young adults (aged 20–39 years).

### National register data

In the Danish Civil Registration System, all residents of Denmark since 1968 have a unique identifier, which is used consistently in all interactions with public institutions and all healthcare services [24]. This allows for linkage between several population-based registers including demographic and healthcare data.

The Danish National Health Insurance Service Register contains data on contacts within the primary healthcare sector in Denmark [25]. The coverage is nearly 100% for medical services, including GPs, other specialist private practitioners and paraclinical examinations. For physiotherapists, services provided on referral from a medical doctor (MD) are registered in this register, while services that are requested and paid for entirely by the user are not.

The Danish Register of Medicinal Product Statistics contains detailed data on all medicines sold in Denmark [26]. Filled prescriptions can be linked to an individual, while over-the-counter sales cannot. In Denmark, paracetamol, ibuprofen, and acetylsalicylic acid can be acquired both over the counter and on prescription, while stronger analgesics and all antimicrobials require a prescription.

The Danish National Patient Register contains detailed data on all in- and out-patient contacts with public and private hospitals in Denmark, including diagnoses of both suspected and confirmed conditions according to the International Classification of Diseases, Tenth Edition [27].

### Risk time and events

We divided each person’s risk time into 24 one-month intervals prior to the date of diagnosis/pseudo-diagnosis.

As events we selected healthcare services likely to be used by patients with non-specific symptoms. In the primary healthcare sector, this included standard consultations and out-of-hours emergency contacts with a GP, visits with other primary sector MDs or physiotherapists, and prescriptions for analgesics or antimicrobials. We also included elective outpatient hospital contacts and emergency room visits.

A given healthcare event could only be registered once per date per person.

### Statistical analyses and model choices

Our main goal was to estimate the number of consecutive months prior to diagnosis where sarcoma patients were more likely than matched comparators to experience a selected healthcare event.

We calculated the hazard ratio (HR) with 95% confidence intervals (CI) for each of the selected healthcare events in sarcoma patients compared to matched comparators for each of the 24 monthly intervals preceding diagnosis. We used stratified Cox regression for recurrent events (Andersen-Gill model) stratified by sex and age group. Baseline hazards were stratified by the matched set, sex, and age group, aligning the analysis with the matching factors and allowing arbitrary baseline differences across these strata while estimating a common exposure effect. No further covariates were included because the design already controlled for the matching factors, and additional post-matching adjustment would risk conditioning on mediators on the causal pathway (e.g., comorbidity or healthcare-seeking), which would attenuate the descriptive pre-diagnostic signal we aimed to show.

Additionally, we wanted to visualize patterns in the HR for each event over the 24 months of prediagnostic observation time, reducing random month-to-month noise without presuming any specific pattern or trend. We used locally weighted regression (LOESS), an unconstrained smoothing method, to draw smoothed curves with a 95% confidence band from the 24 HR estimates of the Andersen-Gill model.[28] As our primary outcome, we report the number of consecutive months prior to diagnosis where the lower bound of the 95% CI for the LOESS curve is greater than one (i.e., HR > 1). For completeness, month-specific HRs with 95% CI from the Cox-models are shown for each monthly interval.

As a secondary analysis, we estimated the HR for each of the selected healthcare events within each of the 24 monthly intervals preceding diagnosis in patients with (M1-stage) and without (M0-stage) metastatic disease at diagnosis compared to their matched comparators.

Data management, analyses, and visualization were performed in RStudio (version 1.4.1717) in a computing environment provided by Statistics Denmark. According to the requirements of Statistics Denmark, we were not allowed to analyze or report any strata of data with fewer than five individuals or specific events. For this reason, some strata are not included in our results, which can be seen in the figures as missing point estimates.

## Results

This study included 1524 individuals diagnosed with sarcoma before 40 years of age (48% females) and 15,047 matched comparators (48% females). Five hundred fifty patients (36%) were diagnosed with a bone sarcoma, 863 (57%) were diagnosed with a soft-tissue sarcoma, and 111 (7%) with unspecified sarcoma. Further characteristics of the sarcoma patients are shown in Supplementary Information 1 – Table A.

**Fig. 1:**
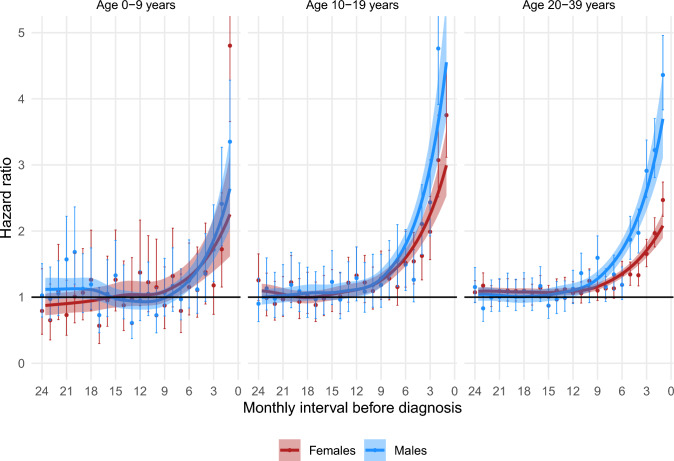
HR of having a consultation with a GP within monthly intervals in the two years preceding a sarcoma diagnosis, stratified by sex and age group

**Fig. 2:**
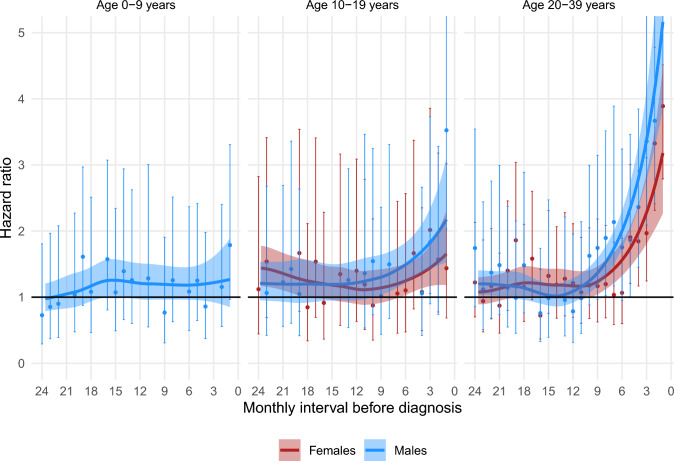
HR of having a consultation with a non-GP primary sector specialist MD within monthly intervals in the two years preceding a sarcoma diagnosis, stratified by sex and age group

**Fig. 3:**
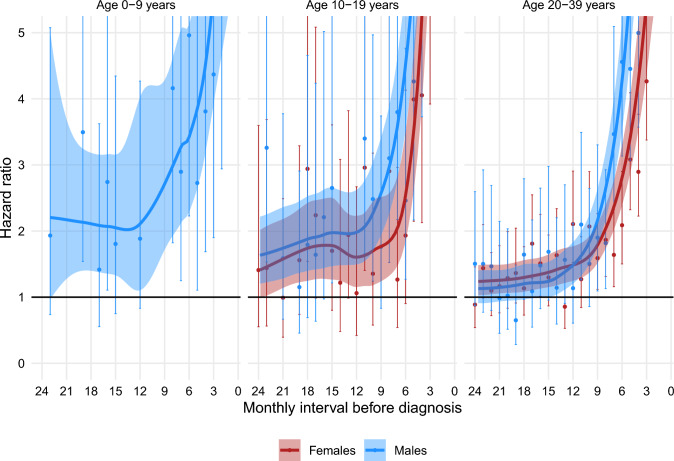
HR of having a contact at a hospital outpatient clinic within monthly intervals in the two years preceding a sarcoma diagnosis, stratified by sex and age group

**Fig. 4:**
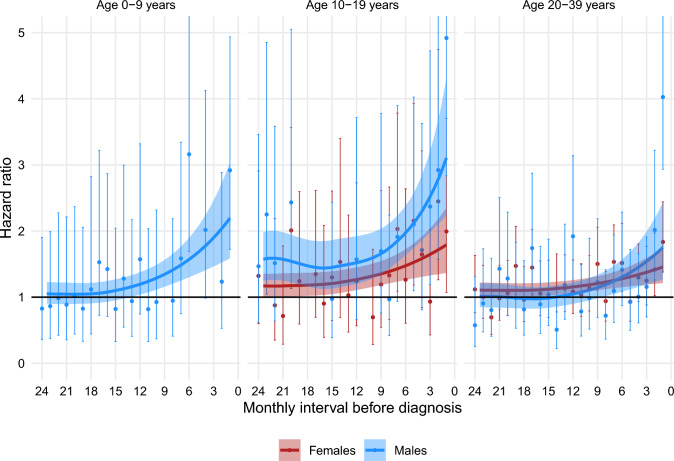
HR of filling a prescription for antimicrobial medication within monthly intervals in the two years preceding a sarcoma diagnosis, stratified by sex and age group

**Fig. 5:**
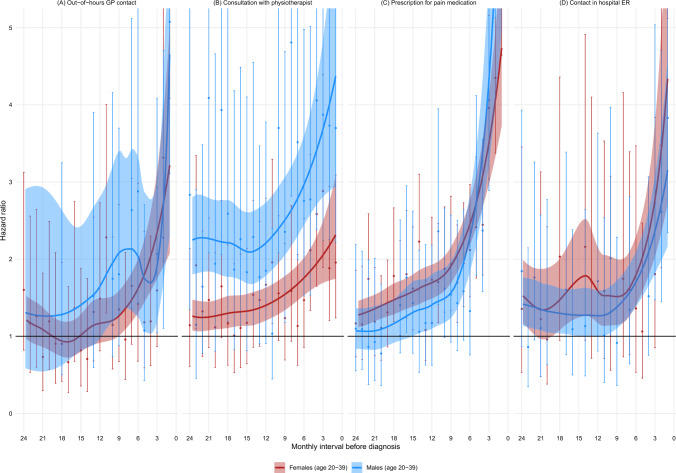
HR of having an out-of-hours contact with a GP, having a consultation with a physiotherapist, filling a prescription for pain medication, or having a contact in a hospital ER, within monthly intervals in the two years preceding a sarcoma diagnosis in patients aged 20–39 years, stratified by sex

Figures 1, 2, 3, 4, 5 show the HRs for specific healthcare events in the 24 months prior to diagnosis for sarcoma patients compared with their matched comparators, stratified by sex and age group. Figures 2, 3, 4 do not show data for pre-adolescent girls because most of the monthly strata had fewer than five events.

### Contacts with a GP

Sarcoma patients had an increased likelihood of having a consultation with a GP compared to matched comparators for 7.5 and 7 months before diagnosis, respectively, in pre-adolescent girls and boys, for 12 and 11 months before diagnosis, respectively, in adolescent girls and boys, and for 15 and 12 months before diagnosis, respectively, in young adult women and men. (Fig. 1.)

### Contacts with other primary sector MDs

Sarcoma patients had an increased likelihood of having a consultation with a non-GP specialist MD compared to matched comparators for 6.5 and 9.5 months before diagnosis, respectively, in adolescent girls and boys and for 9.5 and 11 months before diagnosis, respectively, in young adult women and men. (Fig. 2.)

### Contacts with hospital outpatient clinics

Sarcoma patients had an increased likelihood of having a consultation in a hospital outpatient clinic compared to matched comparators for 24 months before diagnosis in pre-adolescent boys, in adolescents of both sexes and young adult women, and for 14.5 months before diagnosis in young adult men. (Fig. 3.)

### Prescription of antimicrobials

Sarcoma patients had an increased likelihood of filling a prescription for antimicrobial medication compared to matched comparators for 13 months before diagnosis in pre-adolescent boys, for 19.5 and 24 months before diagnosis, respectively, in adolescent girls and boys, and for 16.5 and 8 months before diagnosis, respectively, in young adult women and men. (Fig. 4.)

### Other healthcare events

For the remaining healthcare events, we only had sufficient data for the young adult age group. (Fig. 5.) Compared to matched comparators, young adult sarcoma patients had an increased likelihood of: i) having an out-of-hours contact with a GP for 9 and 12 months before diagnosis, respectively, in women and men, ii) having a consultation with a physiotherapist for 24 months before diagnosis in both women and men, iii) filling a prescription for pain medication for 24 and 15 months before diagnosis, respectively, in men and women, and iv) having a contact at a hospital emergency room for approximately 24 months before diagnosis in both women and men (with slight deviations, which we considered inconsequential).

### Healthcare use and metastatic stage at diagnosis

Seven hundred four patients (46%) were registered with a localized sarcoma (M0), 170 (11%) with metastatic sarcoma (M1), and 171 (11%) with undetermined metastatic status (Mx) at diagnosis. M-stage was not recorded for the remaining 479 patients (31%). Characteristics of the sarcoma patients stratified by recorded M-stage are shown in Supplementary Information 1 – Table B.

**Fig. 6:**
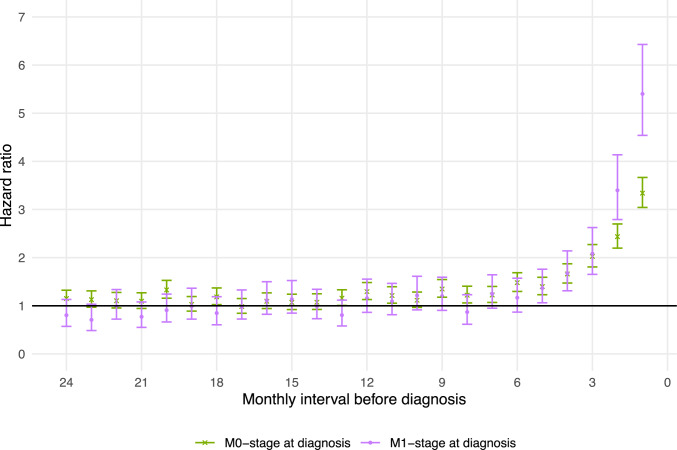
HR of having a consultation with a GP within monthly intervals in the two years preceding a sarcoma diagnosis, for patients with and without metastatic disease at diagnosis

Figure 6 shows the HRs for GP consultations in each of the 24 monthly intervals preceding diagnosis, comparing patients with and without metastatic disease at diagnosis to their respective matched comparators. Only in the two monthly intervals just before diagnosis was there a relative difference between M1- and M0-stage patients, with M1-stage patients having relatively higher HRs. For monthly intervals 3 through 24, M1- and M0-stage patients had similar HRs for GP contacts when compared to matched comparators. Stratifying this analysis by age group, sex, or sarcoma type (bone- vs. soft-tissue sarcoma), respectively, yielded similar results (Supplementary Information 2 – Figures A to C). When stratified by anatomical site of primary tumor, patients with sarcomas of the head and neck appeared to have increased likelihood of contact with a GP for 11 months or longer before diagnosis when compared to their matched comparators, although the data was sparse (Supplementary Information 2 – Figure D).

The HRs for filling pain medication prescriptions was likewise higher for M1- than M0-stage patients only in the two-to-three monthly intervals prior to diagnosis when compared to matched comparators (Supplementary Information 2 – Figure E).

There were no clear relative differences in the HRs for filling a prescription for antimicrobials or having a contact in a hospital outpatient clinic between M1- and M0-stage patients compared to their respective matches (Supplementary Information 2 – Figures F and G).

For the remaining healthcare events, there was insufficient data to compare HRs for M1- and M0-stage patients.

## Discussion

In this population-based study, we examined the use of healthcare services in the two years preceding a sarcoma diagnosis in CAYA. Using stratified Cox and LOESS regression models, we found that sarcoma patients were more likely than matched comparators to experience selected healthcare events for up to 24 months prior to diagnosis. We also compared healthcare use prior to diagnosis in patients with and without metastatic disease at diagnosis and found no differences until shortly before diagnosis.

For most of the selected healthcare events, HRs were increased several months before sarcoma diagnosis (up to two years before diagnosis in several strata) when compared to matched comparators without sarcoma. This strongly indicates a longer interval than previously shown empirically, where symptoms arising from pathobiological processes on the path to a sarcoma led the patient to seek healthcare services. Previous studies identified a period of three to twelve months of increased use of selected healthcare services prior to diagnosis, particularly consultations with a GP [11, 12, 15, 16]. However, these studies were limited in design and scope: Only three studies were population-based [11, 15, 16], none included all early-life sarcomas, none stratified analyses by sex or age, one had observation time of only three months [14], some reported only on consultations with a GP [14, 16], and none included both primary healthcare contacts, prescriptions, and hospital contacts.

Our results suggest that reducing the average diagnostic interval could be possible. Due to the rarity of sarcomas, creating predictive models for population-level screening is not feasible. However, experience from other rare, early-life cancers show, that a national campaign targeted at primary healthcare providers as well as young patients and parents, can lead to shorter diagnostic intervals [10]. Our findings highlight two concrete areas, where future studies and/or campaigns aiming at earlier diagnosis of sarcoma should focus:

Firstly, physiotherapist contacts and prescriptions for pain medication in young adults showed some of the longest durations of consecutively elevated HRs prior to diagnosis. (Due to limited data, we could not conclude on the same events in children and adolescents.) In a previous study, children diagnosed with sarcoma at 0–14 years of age were 6.7 times more likely than matched controls to have had primary care contacts with complaints of “musculoskeletal symptoms” [14]. Our findings suggest that primary sector clinicians could lower their threshold for referral to low-cost diagnostic imaging (i.e., x-ray or ultrasound) in CAYA with persistent musculoskeletal complaints without an obvious diagnosis, particularly in patients requiring several contacts, prescription analgesics or a referral for physiotherapy. As described earlier, this consideration is partly reflected in current guidelines [17–20], however, our findings suggest that more concrete and actionable tools for primary sector clinicians may be needed.

Secondly, the likelihood of having a contact in a hospital outpatient clinic was consistently increased for 24 months prior to sarcoma diagnosis in most strata of sex and age group (14.5 months before diagnosis in young adult men), compared to matched comparators without sarcoma. One previous study in patients older than 15 years found an increased incidence rate ratio of contacts in dermatology, plastic surgery or orthopedic surgery out-patient clinics from 9 months before diagnosis, with no stratification by sex or age group [11]. These findings indicate that outpatient hospital contacts likely play a crucial and understudied role in the diagnostic interval. A study of the diagnostic pathways of teenagers and young adults with cancer in the United Kingdom suggested an increased focus on the transition to, and trajectory within, the secondary healthcare services for reducing diagnostic intervals [29].

To our knowledge, this is the first population-based study to provide empirical evidence comparing the prediagnostic trajectories of CAYA sarcoma patients with and without metastatic disease at diagnosis. Patients with metastatic sarcoma at time of diagnosis were more likely than non-metastatic patients to use healthcare services only in the last 2–4 months prior to diagnosis. Only one previous study in patients older than 15 years compared healthcare service use in cases with localized versus disseminated disease at diagnosis and reported no difference in monthly incidence rate ratios [11]. One possible interpretation of our findings is that the length of the diagnostic interval may not be the primary factor determining whether early-life sarcoma presents with metastasis, at least not beyond the final few months before diagnosis. Rather, sarcomas that are metastatic at diagnosis might have followed a more aggressive pathobiological course, leading to higher disease burden and use of healthcare services in the 2–4 months prior to diagnosis compared to patients with localized disease. Nevertheless, at the individual patient level, shortening the diagnostic interval remains important for reducing the risk of metastatic (as well as locoregional) progression, consistent with the fundamental understanding of cancer as a progressive disease.

In several strata, particularly among young adults, HR values were increased from an earlier time before diagnosis in females than in males. However, in the final months before diagnosis, the increase tended to be steeper in males. This pattern may reflect that young men are more likely than young women to delay seeking healthcare and consequently present at a later stage with more pronounced symptoms. This observed difference likely also reflects a sex-based difference in baseline healthcare utilization in the general population. It is also worth considering whether healthcare professionals might interpret the same symptoms from undiagnosed sarcoma differently in male and female patients, and whether this might cause a sex-based difference in the likelihood that a consultation leads to suspicion of serious illness and a rational diagnostic pathway.

## Strengths and limitations

This first comprehensive, population-based study of healthcare services use prior to diagnosis of sarcoma in children, adolescents, and young adults has several inherent strengths: Firstly, the study is based on population-wide and virtually complete data on both exposures and outcomes across several decades. Secondly, using a descriptive model with (future) case status as the exposure and the occurrence of healthcare utilization as the outcome in a rate-based model allowed us to include participants even if they were not observable for the full 24 months prior to diagnosis. In contrast, previous studies excluded anyone with incomplete observation time [11, 14–16]. Thirdly, stratifying by sex and age group strengthened the study by reflecting clinical differences in health behavior, even if the smaller groups yielded less statistical power.

For 479 patients (31%), M-stage was not recorded, potentially introducing a selection bias in the analysis of healthcare use according to metastatic stage at diagnosis. However, the distribution of sex, age group, and sarcoma type within this subgroup was analogous to the distribution within the entire group of sarcoma patients, suggesting only non-differential lack of classification. (Supplementary Information 1 – Table C).

In Denmark, healthcare services are largely tax-funded, including in the primary sector, and while this does not preclude socio-economic inequalities in healthcare, it does mitigate them. Two population-based studies of the effect of socio-economic parameters on pre-diagnostic use of healthcare services across all pediatric cancers, respectively, showed i) that children from low- and medium-income households had increased likelihood of frequent contacts with a GP within the last three months before diagnosis than children from high-income households, and ii) that for pediatric sarcoma patients specifically there was no effect of socio-economic position on the pre-diagnostic trajectory [30, 31]. While we did not include income or educational level in our models, we did match sarcoma probands and comparators by municipality.

In Danish registers, mild analgesics bought over the counter cannot be linked to an individual. However, sarcoma patients likely use over the counter analgesics more than their peers prior to diagnosis, as pain is a frequent onset symptom. Thus, this limitation of the register data could conceivably lead to an underestimation of the difference between sarcoma patients and their peers.

In settings where the healthcare system is organized and/or used differently than in Denmark, our findings will not be directly transferable, although the overall conclusions should be valid in most high- and upper-medium-income settings. Our findings indicating different prediagnostic pathobiological trajectories of metastatic vs. non-metastatic sarcoma should be valid in other settings.

While conditioning on future events can lead to bias (as the study population by definition are alive for the duration of the study period and thus have a non-existent risk of death and a lower risk of life-threatening events), we believe this potential bias is mitigated by two key factors: Firstly, a young study population in a high-income setting have a low all-cause mortality. Secondly, the most common causes of death for this age group (accidents and acute-onset disease such as infections) would not affect our analyses, as they are unlikely to affect the use of healthcare services several months beforehand. Other life-threatening conditions with slow, insidious onsets are rare in this age group, but we do acknowledge that our study cannot differentiate such a condition from the trajectory of a sarcoma patient. And indeed, we do not expect the effect seen in our study to be unique to sarcomas. Instead, we would expect to observe similar patterns for other life-threatening conditions with insidious onsets. In the clinical setting, the critical step is for a healthcare professional to suspect serious illness and refer for timely and thorough diagnostics. It is far less important whether the clinician specifically suspects a sarcoma.

## Conclusion

Reducing the diagnostic interval may be a viable, low-cost strategy to improve survival from early-life sarcomas, and two areas should be the focus of further studies: Firstly, improving primary sector clinicians’ strategies for selecting CAYA with musculoskeletal complaints for referral to simple, low-cost imaging. And secondly, improving the transition to and trajectory within the secondary healthcare system of CAYA with sarcoma. Future studies should include patients’, caregivers’, and healthcare professionals’ experiences of barriers to earlier diagnosis.

## Supplementary Information

Below is the link to the electronic supplementary material.

Supplementary file1 (PDF 564 KB)

Supplementary file2 (PDF 1219 KB)

## Data Availability

Due to the EU General Data Protection Regulation and Danish national data regulations, the used register data cannot be made available by the authors. Analyses scripts can be made available upon reasonable request.
