# Supplementary material for: Use of healthcare services and prescription medication prior to sarcoma diagnosis in children, adolescents, and young adults in 1997–2020: a population-based cohort study
Source: Cancer Causes Control. 2025 Oct 9;36(12):1963–73. doi: 10.1007/s10552-025-02077-1 (PMC12630154; doi:10.1007/s10552-025-02077-1)
Supplement: Supplementary file 1 — Supplementary file1 (PDF 564 KB) [file 10552_2025_2077_MOESM1_ESM.pdf]

Supplementary Information 1

Use of healthcare services and prescription medication prior to sarcoma diagnosis in children, adolescents, and young adults in 1997-2020: A population-based cohort study

Daniel Thor Halberg Dybdal[1,2], Ólafur Birgir Davídsson[1], Signe Holst Søegaard[1,3], Michael Mørk Petersen[4,5], Ninna Aggerholm-Pedersen[6], Henrik Hjalgrim[1,4,7], Klaus Rostgaard[1], Lisa Lyngsie Hjalgrim[2]

1: Danish Cancer Institute, Danish Cancer Society, Copenhagen, Denmark.

2: Department of Paediatric and Adolescent Medicine, Copenhagen University Hospital Rigshospitalet, Copenhagen, Denmark.

3: Department of Congenital Disorders, Statens Serum Institut, Copenhagen, Denmark.

4: Department of Clinical Medicine, University of Copenhagen, Copenhagen, Denmark.

5: Department of Orthopaedic Surgery, Copenhagen University Hospital Rigshospitalet, Copenhagen, Denmark.

6: Department of Oncology, Aarhus University Hospital, Aarhus, Denmark.

7: Department of Epidemiology Research, Statens Serum Institut, Copenhagen, Denmark.

Corresponding author:

Lisa Lyngsie Hjalgrim

e-mail: lisa.lyngsie.hjalgrim@regionh.dk

Supplementary Information 1 – Table A. Characteristics of all patients with an incident diagnosis of sarcoma before age 40, in the years 1997 through 2020 in Denmark.

	**Females** (n=739)		**Males** (n=785)	
	n	(%)	n	(%)
Age at diagnosis				
0-4 years	30	(4.1)	56	(7.1)
5-9 years	50	(6.8)	56	(7.1)
10-14 years	73	(9.9)	96	(12.2)
15-19 years	96	(13.0)	115	(14.6)
20-24 years	99	(13.4)	86	(11.0)
25-29 years	105	(14.2)	110	(14.0)
30-34 years	116	(15.7)	116	(14.8)
35-39 years	170	(23.0)	150	(19.1)
Histological sarcoma type				
Osteosarcoma	97	(13.1)	102	(13.0)
Ewing sarcoma	88	(11.9)	130	(16.6)
Chondrosarcoma	51	(6.9)	48	(6.1)
Other bone tumor	12	(1.6)	22	(2.8)
Rhabdomyosarcoma	48	(6.5)	88	(11.2)
Non-rhabdomyosarcoma soft tissue sarcoma	384	(52.0)	343	(43.7)
Unspecified sarcoma	59	(8.0)	52	(6.6)
Primary tumor site				
Head and neck	82	(11.1)	87	(11.1)
Trunk wall	187	(25.3)	202	(25.7)
Trunk internal organs	104	(14.1)	62	(7.9)
Extremities	302	(40.9)	360	(45.9)
Other[a]	64	(8.7)	74	(9.4)
Metastatic stage at diagnosis[b]				
M0	348	(47.1)	356	(45.4)
M1	94	(12.7)	76	(9.7)
Mx	71	(9.6)	100	(12.8)
Not recorded	226	(30.6)	253	(32.2)

a: The category *other* includes ICD-O-3 topography codes that do not map to any of the groups above, including ill-defined codes.
b: M-stage in the Danish Cancer Register is the highest TNM M-stage recorded within a four-month period starting from date of diagnosis.

Supplementary Information 1 – Table B. Characteristics of all patients with an incident diagnosis of sarcoma before age 40, in the years 1997 through 2020 in Denmark, stratified by recorded TNM M-stage.

	M0 patients		M1 patients		Mx patients		M-stage missing	
	n	(%)[a]	n	(%)[a]	n	(%)[a]	n	(%)[a]
**All**	**704**	**(46.2)**	**170**	**(11.2)**	**171**	**(11.2)**	**479**	**(31.4)**
Sex								
Female	348	(47.1)	94	(12.7)	71	(9.6)	226	(30.6)
Male	356	(45.4)	76	(9.7)	100	(12.7)	253	(32.2)
Age-group at diagnosis								
0-9 years	97	(50.5)	22	(11.5)	16	(8.3)	57	(29.7)
10-19 years	173	(45.5)	55	(14.5)	36	(9.5)	116	(30.5)
20-39 years	434	(45.6)	93	(9.8)	119	(12.5)	306	(32.1)
Histological sarcoma type								
Bone and cartilage	264	(48.0)	68	(12.4)	48	(8.7)	170	(30.9)
Soft tissue	386	(44.7)	86	(10.0)	104	(12.1)	287	(33.3)
Unspecified sarcoma	54	(48.6)	16	(14.4)	19	(17.1)	22	(19.8)
Anatomical location								
Head and Neck	76	(45.0)	11	(6.5)	29	(17.2)	53	(31.4)
Trunk wall	175	(45.0)	48	(12.3)	37	(9.5)	129	(33.2)
Trunk internal organs	66	(39.8)	29	(17.5)	24	(14.5)	47	(28.3)
Extremities	351	(53.0)	59	(8.9)	63	(9.5)	189	(28.5)
Other[b]	36	(26.1)	23	(16.7)	18	(13.0)	61	(44.2)

a: Percentages are calculated horizontally, i.e., of all patients with a certain characteristic what percentage had a specific M-stage at diagnosis. Rows sum to 99.9 - 100.1 % because of rounding.
b: The category *other* includes ICD-O-3 topography codes that do not map to any of the groups above, including ill-defined codes.

Supplementary Information 1 – Table C. Distribution of sex, age-group and sarcoma type in sarcoma patients with a missing M-stage value and in the entire patient cohort.

	Missing M-stage (n=479)		All patients (n=1524)	
	n	(%)	n	(%)
Sex				
Female	226	(47.2)	739	(48.5)
Male	253	(52.8)	785	(51.5)
Age-group at diagnosis				
0-9 years	57	(11.9)	192	(12.6)
10-19 years	116	(24.2)	380	(24.9)
20-39 years	306	(63.9)	952	(62.5)
Sarcoma type				
Bone and cartilage	170	(35.5)	550	(36.1)
Soft tissue	287	(59.9)	863	(56.6)
Unspecified sarcoma	22	(4.6)	111	(7.3)
