# Supplementary material for: Use of healthcare services and prescription medication prior to sarcoma diagnosis in children, adolescents, and young adults in 1997–2020: a population-based cohort study
Source: Cancer Causes Control. 2025 Oct 9;36(12):1963–73. doi: 10.1007/s10552-025-02077-1 (PMC12630154; doi:10.1007/s10552-025-02077-1)

Supplementary Information 2

Use of healthcare services and prescription medication prior to sarcoma diagnosis in children, adolescents, and young adults in 1997-2020: A population-based cohort study

Daniel Thor Halberg Dybdal[1,2], Ólafur Birgir Davídsson[1], Signe Holst Søegaard[1,3], Michael Mørk Petersen[4,5], Ninna Aggerholm-Pedersen[6], Henrik Hjalgrim[1,4,7], Klaus Rostgaard[1], Lisa Lyngsie Hjalgrim[2]

1: Danish Cancer Institute, Danish Cancer Society, Copenhagen, Denmark.
2: Department of Paediatric and Adolescent Medicine, Copenhagen University Hospital Rigshospitalet, Copenhagen, Denmark.
3: Department of Congenital Disorders, Statens Serum Institut, Copenhagen, Denmark.
4: Department of Clinical Medicine, University of Copenhagen, Copenhagen, Denmark.
5: Department of Orthopaedic Surgery, Copenhagen University Hospital Rigshospitalet, Copenhagen, Denmark.
6: Department of Oncology, Aarhus University Hospital, Aarhus, Denmark.
7: Department of Epidemiology Research, Statens Serum Institut, Copenhagen, Denmark.

Corresponding author:
Lisa Lyngsie Hjalgrim
e-mail: lisa.lyngsie.hjalgrim@regionh.dk

Figure A. HR of having a consultation with a GP within monthly intervals in the two years preceding a sarcoma diagnosis, for patients with and without metastatic disease at diagnosis – stratified by age-group.

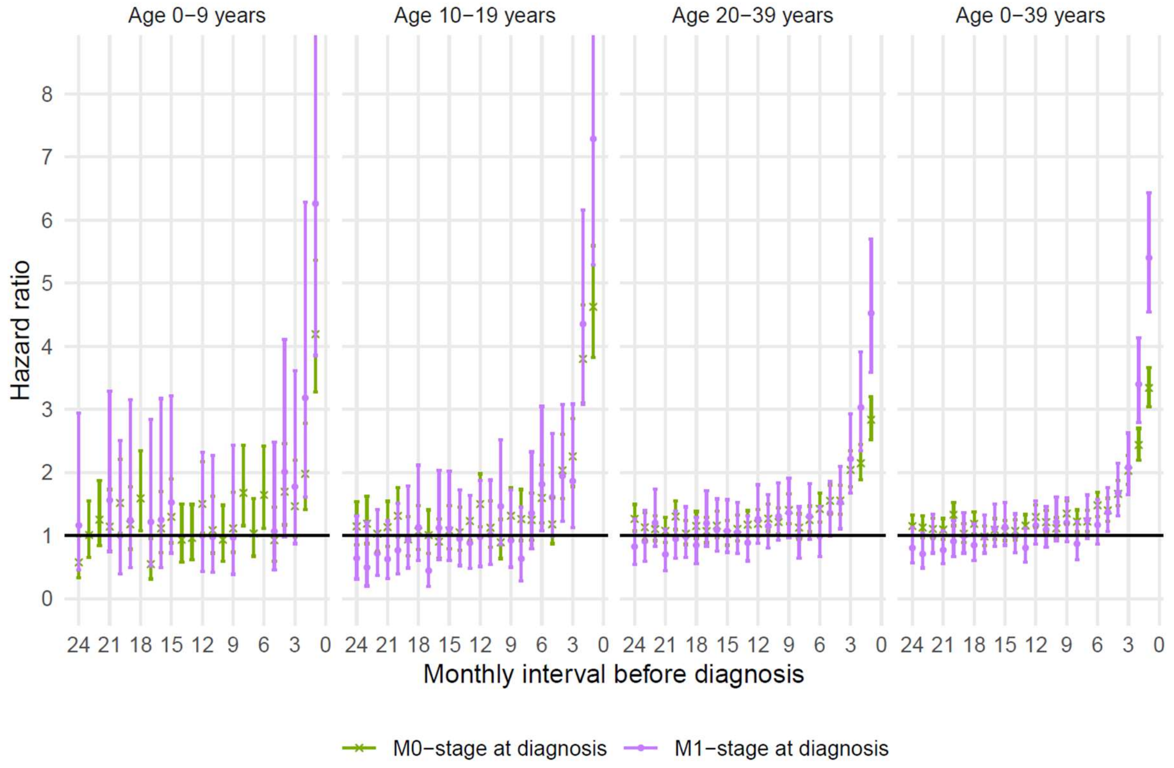

Figure B. HR of having a consultation with a GP within monthly intervals in the two years preceding a sarcoma diagnosis, for patients with and without metastatic disease at diagnosis – stratified by sex.

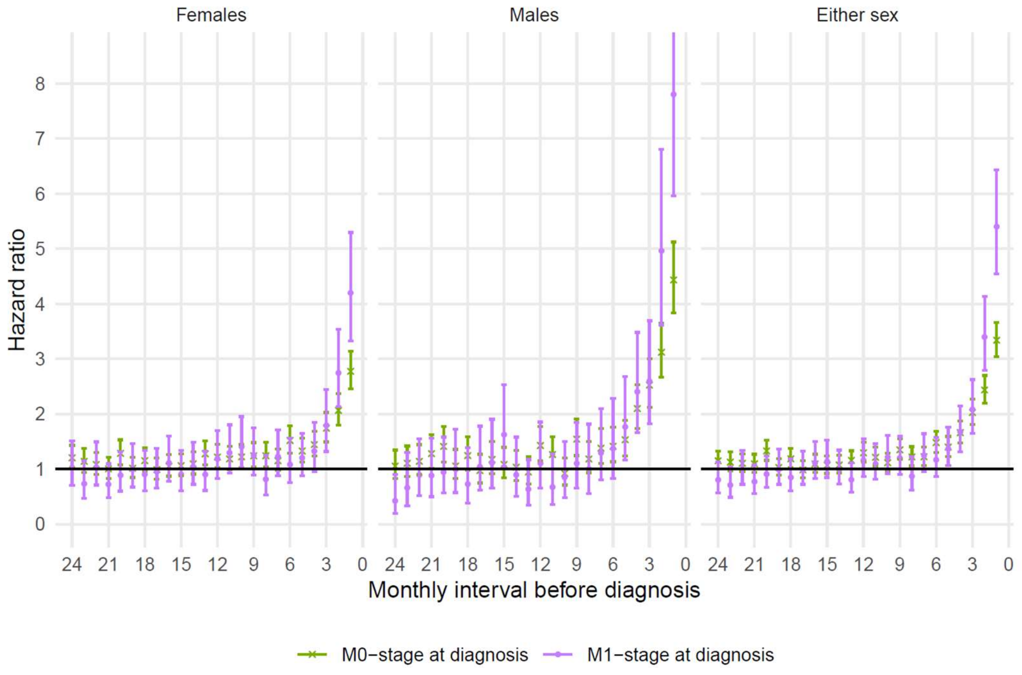

Figure C. HR of having a consultation with a GP within monthly intervals in the two years preceding a sarcoma diagnosis, for patients with and without metastatic disease at diagnosis – stratified by sarcoma type.

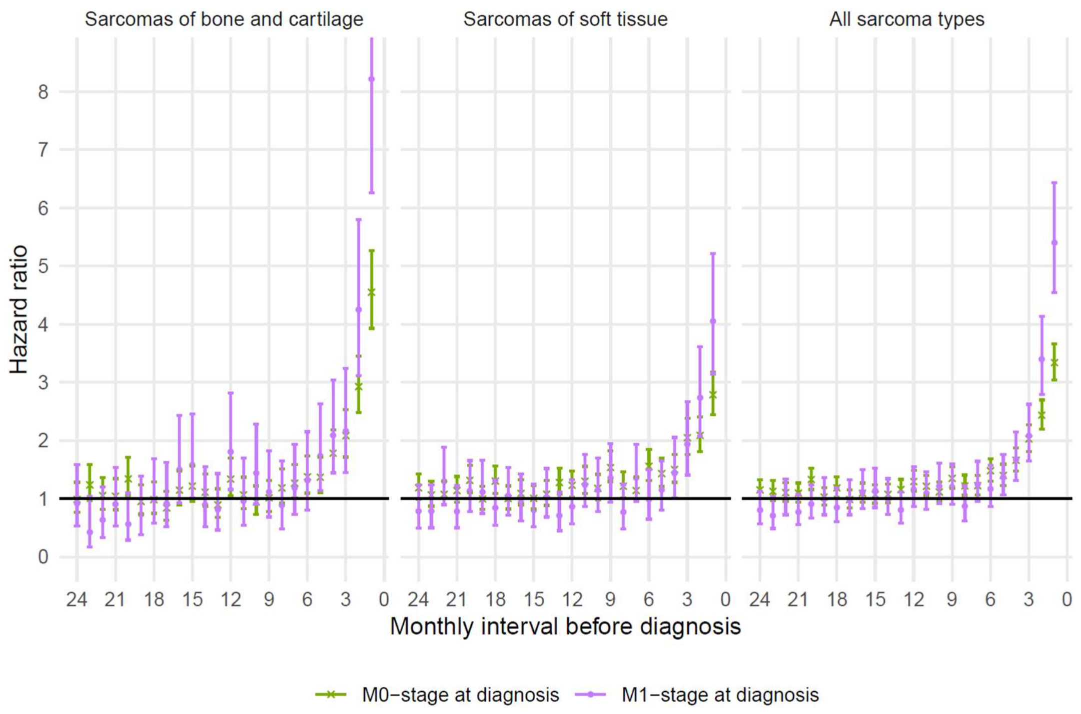

Figure D. HR of having a consultation with a GP within monthly intervals in the two years preceding a sarcoma diagnosis, for patients with and without metastatic disease at diagnosis – stratified by anatomical location of primary tumor.

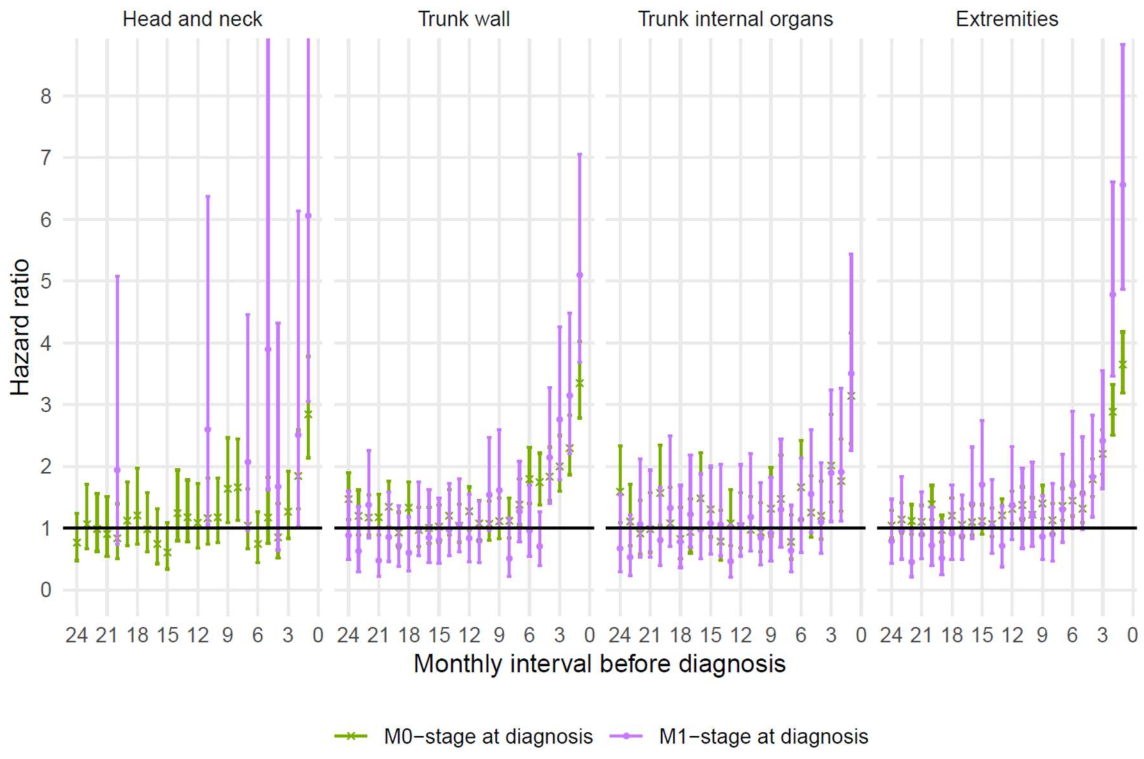

Figure E. HR for filling pain medication prescriptions within monthly intervals in the two years preceding a sarcoma diagnosis, for patients with and without metastatic disease at diagnosis.

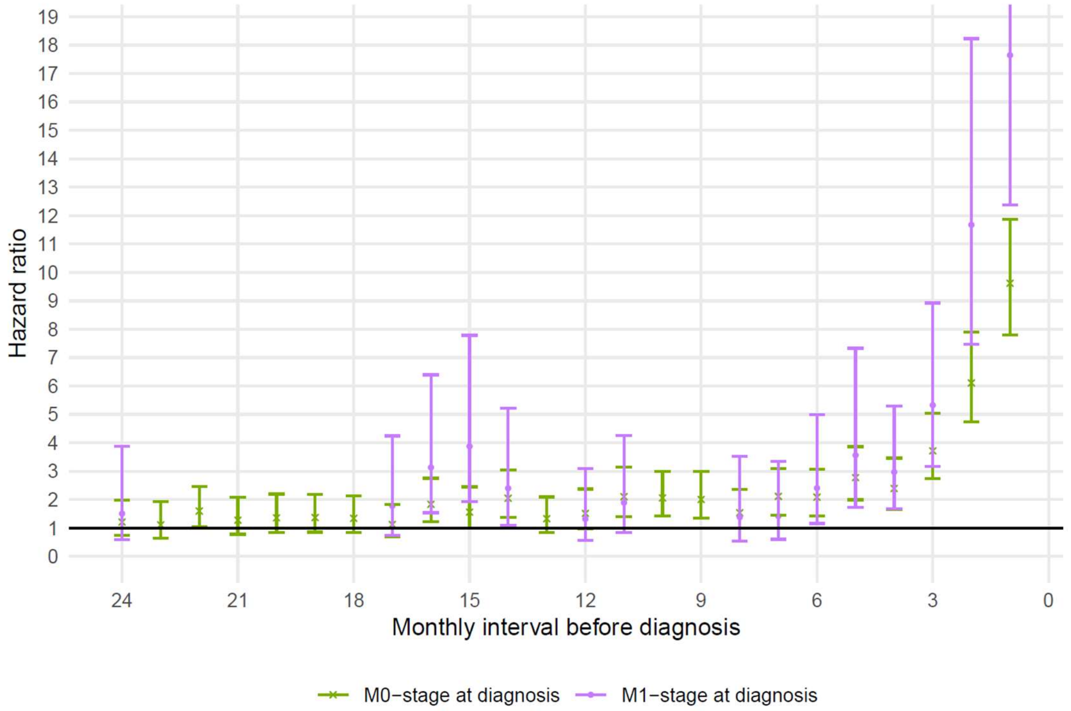

Figure F. HR for filling antimicrobial medication prescriptions within monthly intervals in the two years preceding a sarcoma diagnosis, for patients with and without metastatic disease at diagnosis.

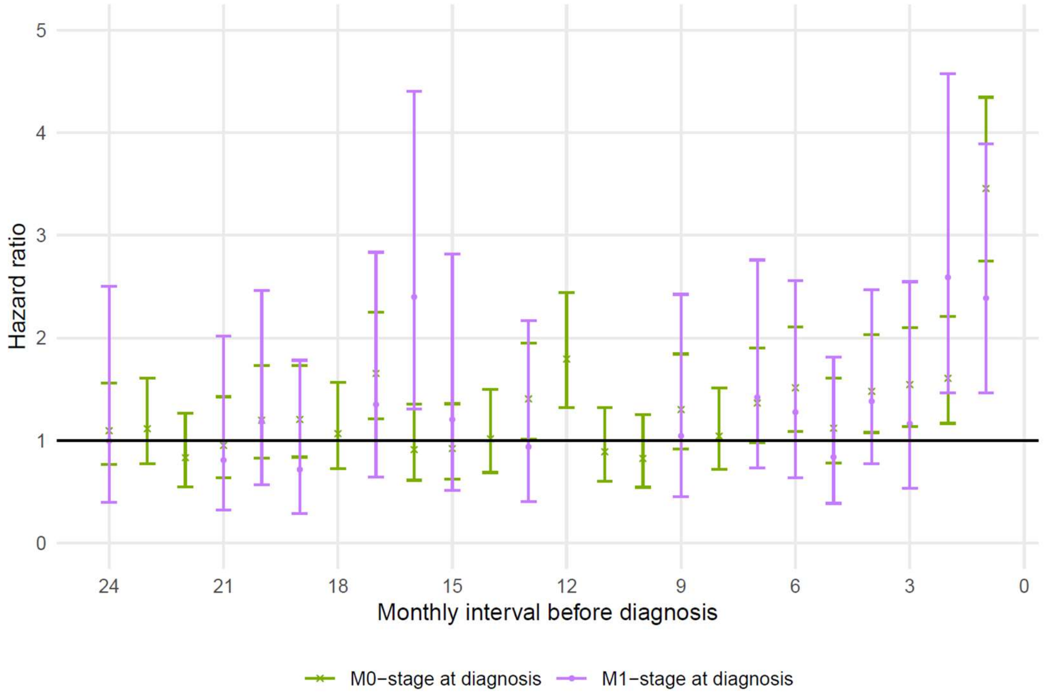

Figure G. HR for contacts in a hospital outpatient clinic within monthly intervals in the two years preceding a sarcoma diagnosis, for patients with and without metastatic disease at diagnosis.

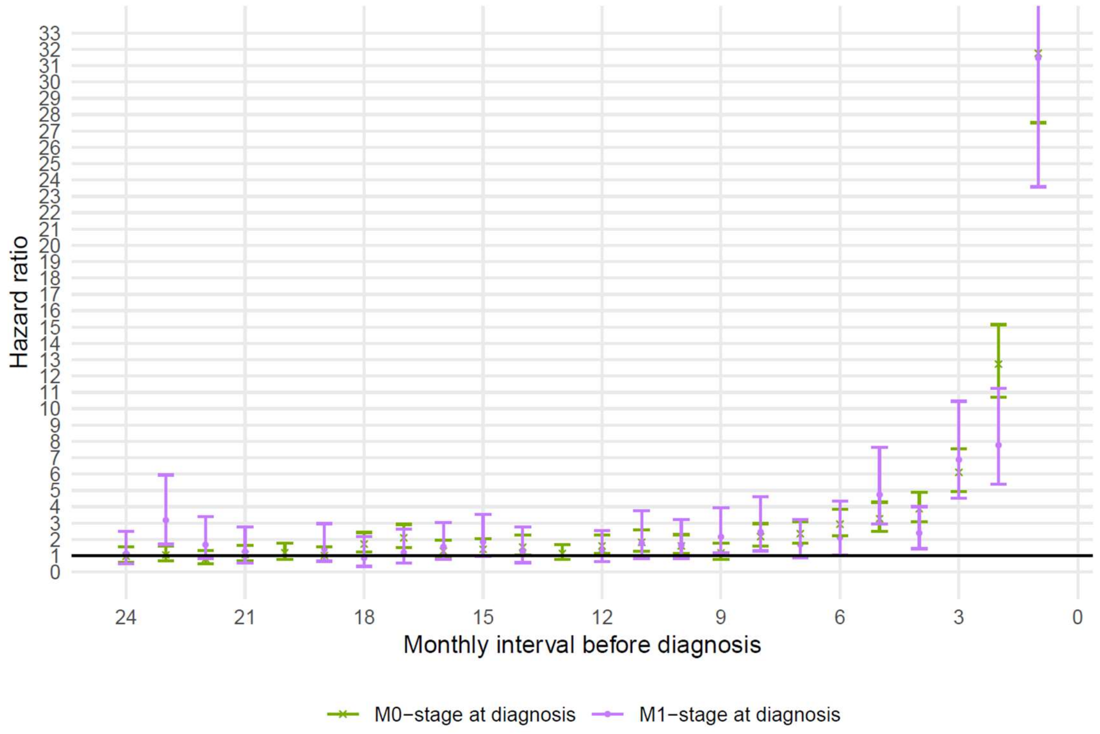

Supplement: Supplementary file 2 — Supplementary file2 (PDF 1219 KB) [file 10552_2025_2077_MOESM2_ESM.pdf]
